# Female mice exhibit less overall variance, with a higher proportion of structured variance, than males at multiple timescales of continuous body temperature and locomotive activity records

**DOI:** 10.1186/s13293-022-00451-1

**Published:** 2022-07-23

**Authors:** Benjamin Smarr, Lance J. Kriegsfeld

**Affiliations:** 1 Department of Bioengineering, UC San Diego, San Diego, CA USA; 2 Halicioğlu Data Science Institute, UC San Diego, San Diego, CA USA; 3 Department of Psychology, UC Berkeley, Berkeley, CA USA; 4 Department of Integrative Biology, UC Berkeley, Berkeley, CA USA; 5 The Helen Wills Neuroscience Institute, UC Berkeley, Berkeley, CA USA

**Keywords:** Sex differences, Biological rhythms, Mathematical biology

## Abstract

Despite recent work demonstrating that female rodents and humans do not show greater variance in behavior and physiology than males due to ovulatory cycles, many researchers still default to using males in their investigations. Although government funding agencies now require inclusion of female subjects where applicable, the erroneous belief that the study of males reduces overall data variance continues to result in male subject bias. Recently, we reported the first direct experimental refutation of this belief by examining continuous body temperature and locomotor activity in male and female mice. These findings revealed that males exceeded female variance within and across individuals over time, showing greater variance within a day than females do across an entire estrous cycle. However, the possibility remains that male variance within a day is impacted by ultradian rhythms, analogous to the influence of infradian estrous cycles on female variance, and both sexes show predictable, structured variance across the day. If structures underlying variance can be predicted, then the variance can be statistically accounted for, reducing experimental error and increasing precision of measurements. Here we assess these continuous body temperature and activity data for the contributions of structured and unstructured variance to overall variance within and across individuals at ultradian, circadian, and infradian timescales. In no instance do females exceed male variance, and in most instances male variance exceeds female variance. Additionally, more female variance is accounted for by temporal structure. In conclusion, even when estrous cycles are not controlled for, females show less variability than males, and this advantage can be further capitalized upon by inclusion of known temporal patterns to control for previously unknown but structured sources of variance.

## Highlights

- Female mice do not show more overall variance in temperature or activity than do males.
- When measuring rodents, phase of the daily rhythm accounts for a substantial amount of the overall variance.
- Female mice show less unstructured variance than do male mice, and for both sexes, time of day is the biggest influence on structured variance, with estrous cycle variance accounting for as little as 3% overall.
- Many analytic approaches allow for quantification of similarity or predictability in biological time series data, and should be adopted more broadly to improve measurement precision for subjects of any sex.
- Sex differences exist in mean, variance, variance composition, and rhythm composition for most measures. The evidence presented here that female subjects are less variable overall than males should encourage inclusion of Sex as a Biological Variable in preference to only including one sex in a study.

## Introduction

Despite mandates from the National Institutes of Health (NIH) to include sex as a biological variable in study design [1], many investigators remain resistant to the use of female subjects in research [2, 3] due to the belief that un-staged ovulatory cycles give rise to unacceptable, high levels of variance in experimental measures. This exclusion has resulted in myriad real costs to female patients, including persistent increased risk of side effects from many pharmaceuticals [4, 5], and delays in care and decreased care efficacy for cardiovascular disease [6]. Recent meta-analyses [7–10] and direct comparisons in mice [11] have provided empirical rebuttal of the conjecture that females (rodent and human) are in fact more variable. However, these analyses also point to a lack of standardized tools for assessing differing sources of variance in biological time series data.

If males and females have comparable variance across measures, and ovarian cycles account for much of the variance observed in females, then females may show more structured variance than males or structure may occur at different timescales between the sexes. Unstructured (“random”) variance implies that knowledge about the system cannot be applied to reduce the effective uncertainty surrounding a given measure (e.g., white noise in a signal, or Brownian motion affecting precise location measurements of particles), whereas structured variance implies that patterns explain away (“reduce”) some of the overall variance, reducing the effective uncertainty around those measurements to which structures apply (e.g. knowing the Earth orbits the sun regularly allows us to predict winter in the northern hemisphere, whereas random samples of temperature with respect to time would only reveal that sometimes the northern hemisphere is cold, and sometime hot, so that almost any temperature might seem “potentially normal” for a random sample from the northern hemisphere). In biological systems, phase angle of ultradian and circadian rhythms are structures that explain some of the overall variance of physiological measurements; uncertainty around a specific phase, as in midnight, can be reduced in comparison to the overall variance because midnight variance is more accurately drawn by comparison to other measures taken at midnight, without including variance from measures taken at noon, which are known to fall in a different distribution as a result of being at the opposite circadian phase. As a concrete example of this principle, we recently showed that fevers associated with COVID-19 were difficult to detect when single temperatures were taken at unspecified phases. However, phase-specific comparisons made possible by continuous measurement from wearable devices made fevers not only clearly detectable, but also predictable in many cases. Loss of important signals such as fever onset from COVID-19 provide a life and death illustration of the importance of reducing structured variance to gain precision when possible. Similar life and death errors have resulted from the exclusion of female subjects, highlighting further the need for quantitative methods to assess structured and unstructured variance specific to males and females as separate populations.

Currently, there are not standard approaches to assessing the relative contribution of structured and unstructured variance to biological time series data. Here, we apply multiple approaches to determine the extent of structured versus unstructured variance in previously-published temperature and activity data from male and female mice. Additionally, these approaches are used to compare contributions of structure to variance at different timescales in males and females variance.

## Methods

### Data gathering

Data published previously [11] were re-analyzed, with no new animal experiments conducted. Briefly, data were previously generated using 13 male and 13 female 8–12 week old BALB/c mice using 1-min resolution recordings of body temperature (CBT) and locomotor activity (LA). CBT and LA were gathered using a G2 minimitter (Starr Life Sciences Co., Oakmont, PA), implanted several weeks previously in the intraperitoneal cavity and secured to the inside of the abdominal wall to maintain consistency. Animals were not handled or otherwise disturbed during the 14-day period of data assessed here, other than weekly cage changes.

### Cumulative error, and static vs. dynamic error rates

To quantify the cost of interpreting biological measurements made without knowledge of cyclic context, we define the concept of “cumulative error rates”. Such errors represent the amount of distance from the expected measurement should samples be accumulated randomly with respect to time (that is, with the assumption that all error is random error arising from entirely unstructured variance). The cumulative error rate is the rate of the accumulation of erroneous measurements, defined as those measures more than one standard deviation (SD) from the comparative mean. To assess sex differences in cumulative error rates over time, we defined (mathematically operationalized) two kinds of error, based on two definitions of the “comparative mean”: (1) Static error; and (2) Dynamic error. (1) Static error was defined as the distance beyond one SD from the mean for each measurement, where mean and SD were calculated using all individuals (i.e., pooled males and females) and all timepoints (i.e. also pooling time), resulting in a single comparative mean and SD for all individual measurements. (2) Dynamic error was calculated analogously, but with a mean and SD calculated across all individuals, but separately for each min of data, resulting in a time series of mean and SD pairs. By these definitions of error, a value within 1 SD of the population mean has an error value of 0, while a measure 3 SD from the mean has an error value of 2: $\left( {\left( {\left( {measurement{-}mean} \right) \, / \, SD} \right){-}1} \right).$

### Aligning vs. staggering estrous cycles

Female data contain three estrous cycles per individual (4 days per cycle per individual, with 1 day of additional buffer on each end, for a total of 14 days per individual). To test the impact of estrous cycles on static and dynamic error measurements, female data were arranged in two extremes of interindividual alignment. Maximum alignment was achieved by aligning each female so that day 2 of 14 was a day of estrous, so that the subsequent 3 cycles were on the same phase for each individual in each day. To simulate the error from measurements of females for whom cycle phase in unknown, female data were staggered to maximize misalignment. This was achieved by taking the matrix of aligned female data (1 column per minute, 1 row per individual) and advancing each subsequent row by 1440 min (1 day) relative to the previous row. The result is 4 sets of 3 individuals aligned to each other, but between 1 and 3 days out of alignment with the other sets every day. This allowed us to simulate maximum entropy of phases per unit time as an antithesis to maximum alignment. When necessary for comparison, male data underwent the same misalignment process; this was as a control for the manipulation, as male data do not have estrous days to create an aligned state.

### Dynamic time warping

**Fig. 1:**
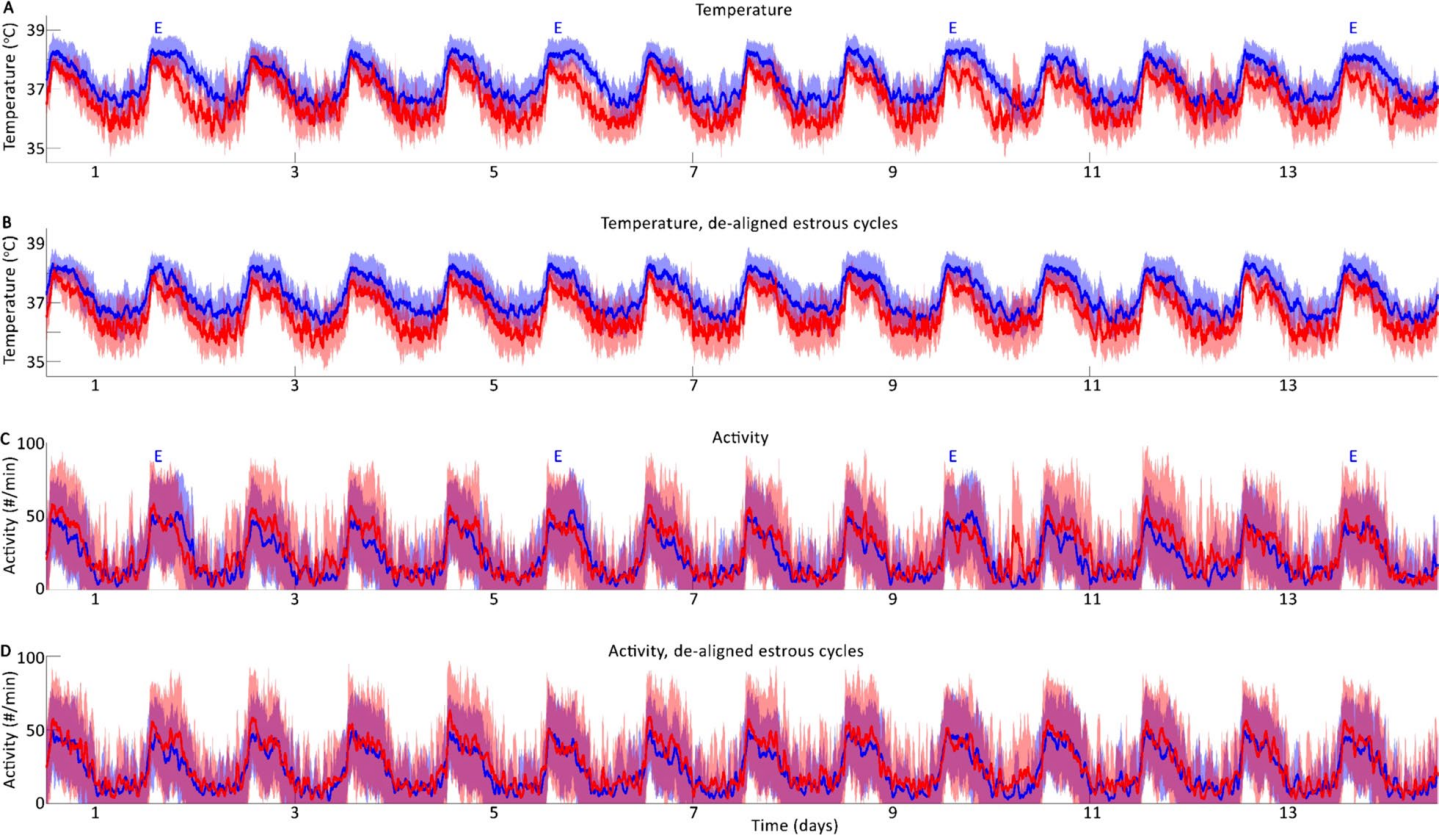
Core body temperature **A**, **B** and locomotor activity (**C**, **D**), for males (red) and females (blue), presented as mean ± SD. Some analyses use a data set in which females are aligned by days of estrous (“E”s; **A**, **C**), and some use a data set in which each individual (males and females) has been delayed 1 day relative to the previous individual in its group (**B**, **D**), thereby maximally de-aligning females to insure that interindividual structure is not conferred to the female group by way of predictable, 4-day ovulatory cycles

Dynamic time warping (DTW) was run using the Matlab command “dtw”, with no incremental boundary set (meaning warping could potentially be as large as the length of the data set, with the trade-off that no arbitrary limits were imposed on the process). DTW applies an incremental offset to the X values of one signal, with a function to minimize the distance between the two signals following the warping of the first signal (i.e. the Y-values of the signals are not changed, but can be pushed around so as to align previously near but misaligned peaks, etc.). This approach does not yield a comparison to each individuals’ average day, but instead provides an amount of difference (warping distance) between each pair of days’ time series data for each individual. Averages were calculated on data with days staggered to minimize cycle alignment across females, as described in “Methods: Aligning and staggering estrous cycles,” and in Fig. 1. This choice allowed us to avoid artificially deflating female average distance per day due to a increased similarities observed across individuals’ days of estrus (data not shown).

### Data cleaning

Data were cleaned as follows: For CBT, outlier values below 35 were set to 35, and all points greater than 3 standard deviations (SD) of the mean were set to three SD from the mean (plus or minus, as appropriate). For activity, the correction was only applied in the positive direction so that activity counts of “0” were not inflated. Wavelet analysis was run using in-house code for continuous wavelet transformation (CWT) modified from the updated “Jlab” toolbox developed by Dr. J Lilly, and further modified and provided by Dr. Tanya Leise (described in [12]), using the morse wavelet (*β* = 5, *γ*= 3). Wavelet coherence was run using the built in Matlab function “wcoherence”, described in [13].

### Data processing and statistical analysis

Data were processed and visualized using Matlab 2018a. Code and raw data are available upon request, or at the author’s UCSD website (smarr.ucsd.edu). CWT bands were defined as the maximum wavelet power (the product of the amplitude of the wavelet and the time series at a given moment) per minute for the range of periodicities from 23 to 25 h (“circadian power band”) and 1–3 h (“ultradian band power”). Each individuals’ data were transformed and analyzed separately, and population averages used to assess changes in band power distribution by sex are presented as both means and medians so as not to constrain interpretations to those appropriate under assumptions of normal distributions within these populations. For Fig. 1C, CWT was run across the ultradian wavelet band power for each individual, and circadian band of this second order wavelet extracted to assess circadian modulation of ultradian periodicity (i.e., circadian power is extracted from the CWT band of the ultradian power already extracted from the original signal’s CWT, providing a measure of how strong the time of day modulates the power of the ultradian rhythms).

**Fig. 2:**
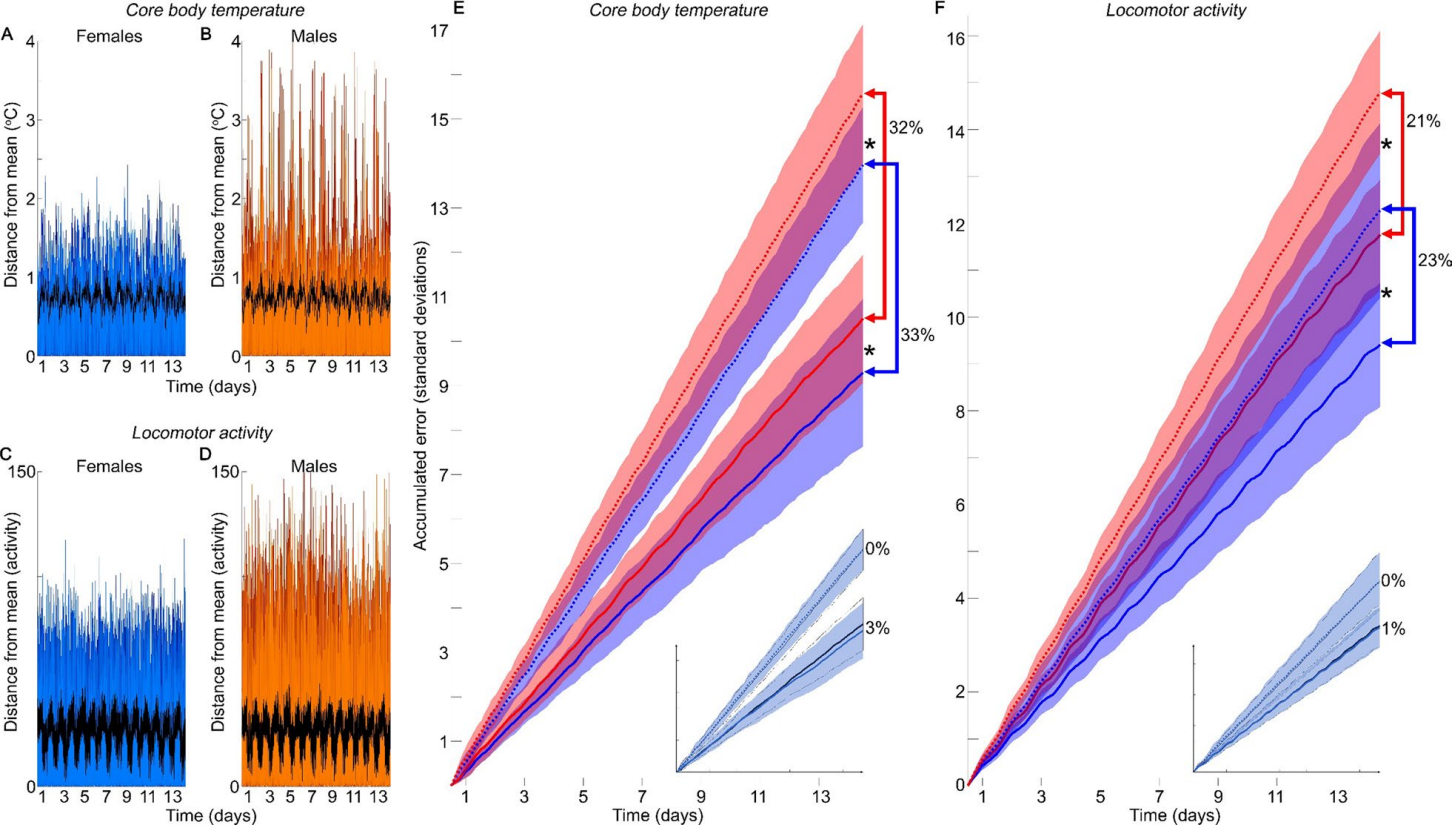
The estrous cycle does not contribute substantially to overall variance, of which males have more than females. Static error for all individual females (A, C, blue tones) and males (**B**, **D**, in red tones); female days arranged to provide minimum possible alignment across individuals’ estrous cycles; Y = 1 = Static SD; black line = dynamic SD. Males have higher variance than females in both temperature **A**, **B** and activity (**C**, **D**). Sex affects the accumulation rate of static and dynamic errors across the 14 day data window (**E**, **F**). Males have a higher cumulative error than do females, even when estrus is not aligned for females (**E** temperature, **F** activity); males in red, females in blue; line is intra-sex mean, filled areas are intra-sex SD of accumulated error; units in static SD of the population. Roughly one third of the error can be eliminated (blue and red arrow brackets) by comparison to a population dynamic baseline (solid lines) to a static baseline (dotted line). Insets: The estrous cycle adds additional structure, so that staging females (aligning individuals by estrous cycle) further reduces the accumulated errors for females, when comparing to a dynamic baseline, by 3% for temperature and 1% for activity; 0% change in males for the same realignment. *Indicates significant difference. See “Results” section for further details

Statistics were run in Matlab 2018a. Comparisons are non-parametric Wilcoxon rank sum tests (presented as *p*-value) and analysis for Fig. 2 uses a Kruskal Wallis test of the last 1 h of cumulative error scores (presented as χ^2^ and *p*-value). Findings were considered statistically significant when *p* < 0.05.

## Results

### Analysis of the role of sex in the rate of accumulated error

Using the present data set, we have previously shown that that males had higher variance than females in CBT and LA within and across individuals [11]. These data (Fig. 1) were reanalyzed here to assess the contributions of different timescales and structures to variance observed.

If variance is unaffected by sex, then one would expect that measurement errors would accumulate overtime at the same rate for males and females, or that sex does not impact precision of a given measurement. We did not find this to be the case. Here we assessed this using two kinds of error: 1) Static error; and 2) Dynamic error (see “Methods” section). Cumulative static error would be expected to differ from cumulative dynamic error if a portion of the variance were structured in time, leading to changes over time that reduce overall error (i.e., individual measures change over time in similar, non-random ways). Importantly, initially analyses used female data that had been staggered so that estrous days were maximally unaligned across animals, so that no 4-day pattern (i.e., the period of a mouse estrous cycle) is responsible for the error structure in the both-sexes mean and SD. A visual depiction of this strategy is presented in Fig. 1.

Raw data presented as distance from the overall mean revealed that males displayed higher variability than females for both CBT (Fig. 2A, B) and LA (Fig. 2C, D). Quantification of the cumulative error for CBT and LA (Fig. 2E, F, respectively) revealed that males accumulated significantly more error over time than did females (Static, CBT: *χ*^2^ = 401, *p* = 3 × 10–89; Static, LA: *χ*^2^ = 580, *p* = 4 × 10^–128^; Dynamic, CBT: *χ*^2^ = 282, *p* = 3 × 10^–63^; Dynamic, LA: *χ*^2^ = 791; *p* = 5 × 10^–174^). Cumulative static error rose more quickly than did cumulative dynamic error, confirming that some of the variance in the data was structured in time; comparison to the dynamic baseline was thereby confirmed to reduce measurement uncertainty. Note that error reduction was greater in CBT than in LA when a dynamic baseline was used (females *p* = 0.007, males *p* = 0.006), suggesting that CBT showed more structured variance across time than did LA. When these analyses were re-run on data in which females were staged (data aligned by days of estrus), the error was reduced further (3% for temperature, 1% for activity—Fig. 2E, F insets), confirming that estrous cycles contributed a small but structured amount of variance to the overall mean and SD of the population data.

### Examination of amount of change within and across days, including ultradian structure, across individuals

**Fig. 3:**
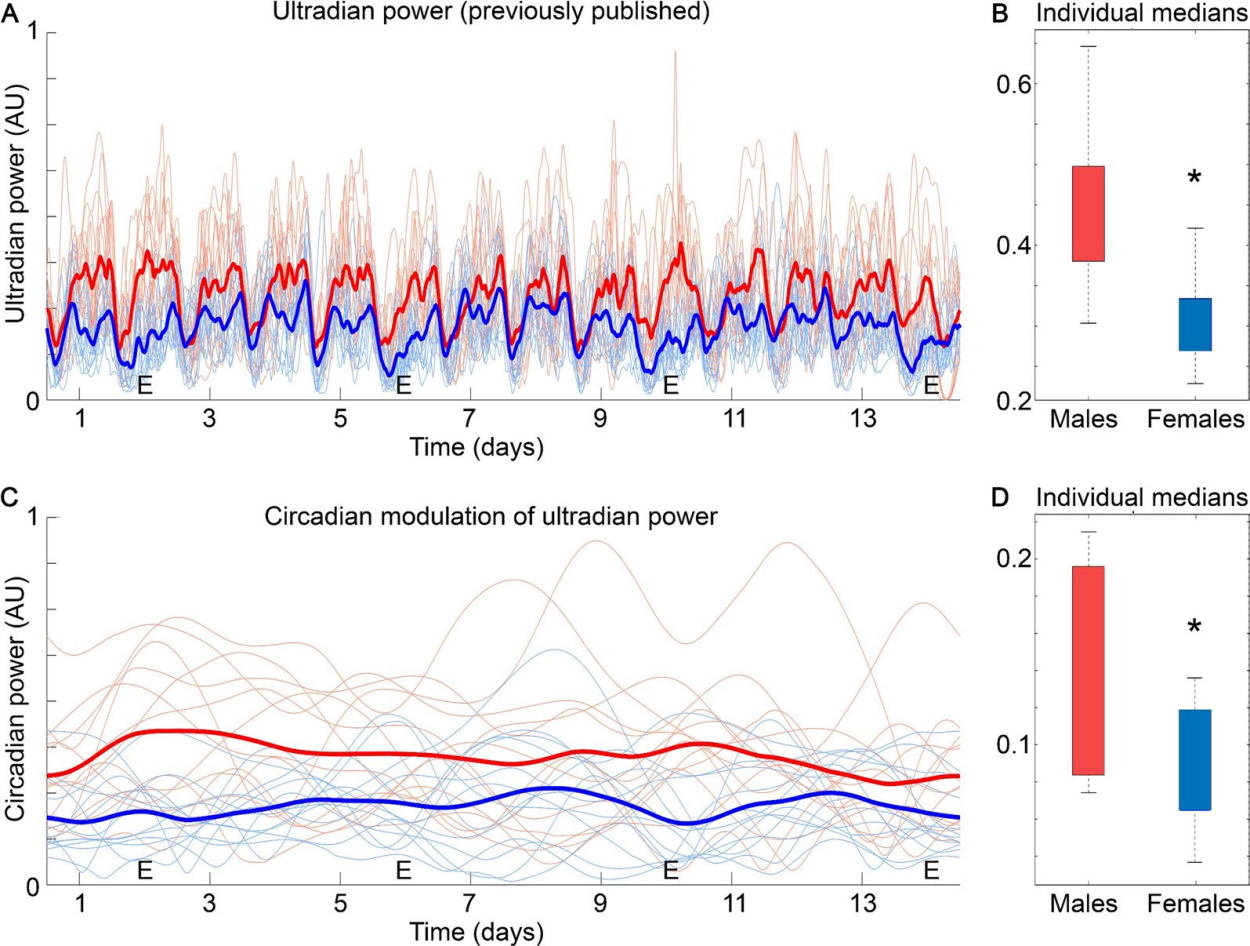
Previously published temperature analyses **A** found that male mice (red lines—thick line is mean) show higher amplitude changes in ultradian rhythms (as determined by wavelet-based frequency band isolation—see “Methods” section) across time than females (blue). Here females are aligned by their 4-day estrous cycles (“E” marks days of estrus). Building on these analyses, males also have a higher median ultradian power, and inter-individual range of medians (**B**), than do females. Consistent with this finding, wavelet-based isolation of circadian modulation of ultradian power shown in **A** (**C**) reveals that males have greater circadian modulation of ultradian rhythms than do females. In addition to having higher median power **D**, males also show greater inter-individual variance than do females for both median ranges. *Indicates significant difference. See “Results” section for further details

Our previous findings using the present data set found that males exhibited larger variance across the day than did females, but the structure of that variance was not assessed [11]. As previously reported [11], males showed a higher amplitude of change in the CWT frequency power of their ultradian temperature rhythms (Fig. 3A). Further analysis revealed that males also showed a higher median power overall (Fig. 3B; *p* = 3 × 10^–4^), as well as a higher median power of the circadian modulation of ultradian power (Fig. 3C, D; *p* = 0.01). These findings indicate that males exhibited a greater change resulting from higher amplitude ultradian rhythms in body temperature, and also resulting from higher amplitude modulation of these ultradian rhythms across the day, than did females.

### Analysis of structure within individuals within a day

The analyses presented so far suggest that male mice exhibit more variability, less of which is structured, across days, than do female mice. However, it is possible that males show more structure within a day than females. Ultradian rhythms are not perfectly alignment day to day [11, 14, 15], so we chose two methods to assess self-similarity across days: comparison to a personal mean day, and day-to-day difference calculated by dynamic time warping (DTW).

**Fig. 4:**
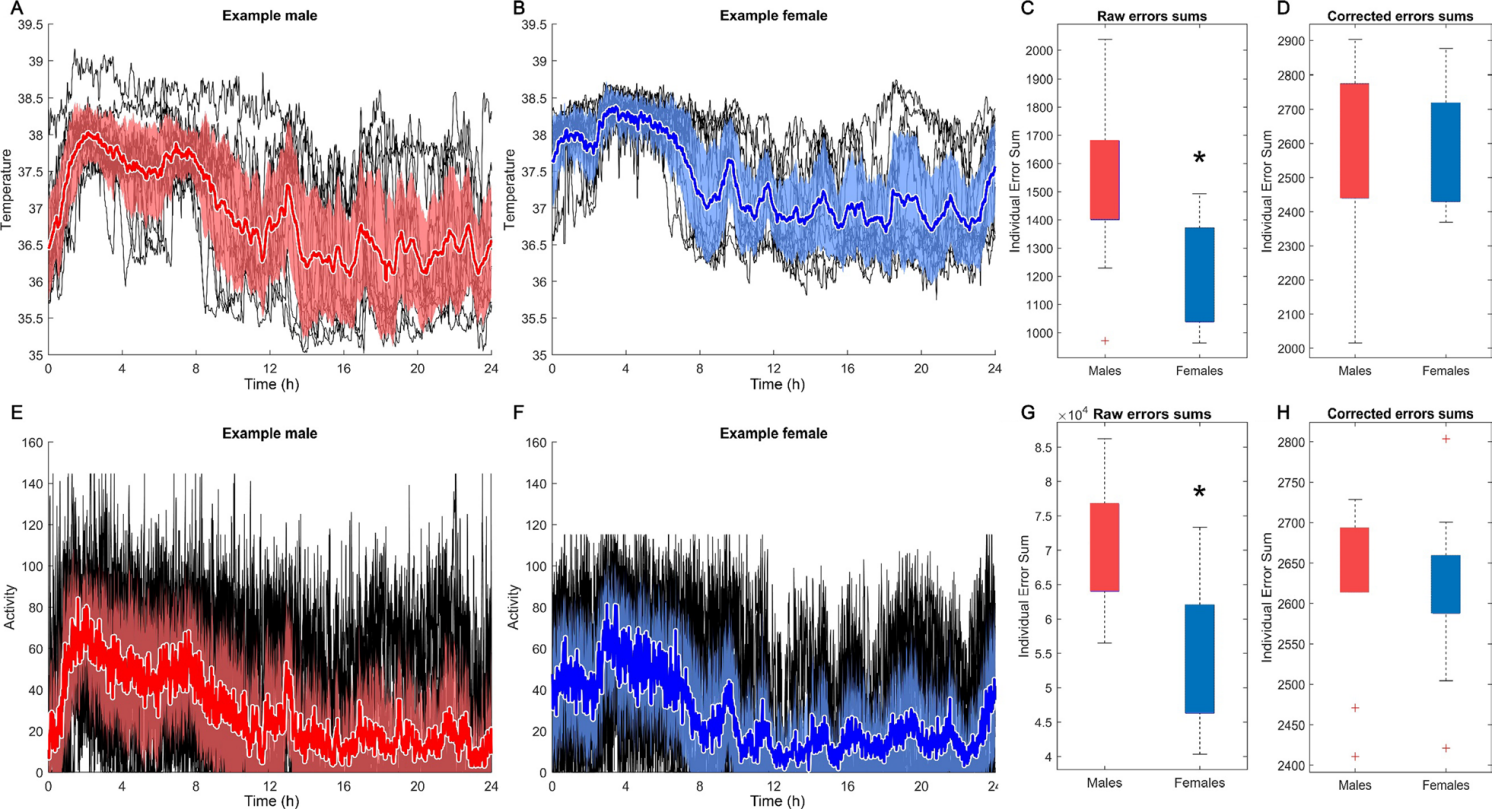
Males did not show more structure within days than females. Mean and SD (thick line with shaded surround) of temperature **A**, **B** and activity **E**, **F** of one male (red) and female (blue) across 24 h, overlaid on 14 days for the same individual (black lines underneath color). Any measurement more than 1 SD from the mean is defined as error. Error summed across 24 h allows comparison of structured variance by sex within a day. Males show significantly higher within individual, within-day error than females (**C**, **G**). Structure of the variance appeared similar across sexes; there was no difference between the sexes once each individual’s error was divided by that individual’s mean SD (**D**, **H**). This finding demonstrates that males did not have sufficient structure within a day to make them overall less variable if within-a-day structure is accounted for in this way. However, the amount of error was proportional to the SD in both sexes, suggesting that while males were more variable overall, they did not have less structure within a day than did females, by this approach. *Indicates significant difference. See “Results” section for further details

We first examined within-a-day structure by constructing a mean daily profile for each individual, and calculating dynamic cumulative error in the same way as in the previous section, comparing each animal’s individual days to their own mean day, both for temperature and activity. By these analyses, males had a higher rate of cumulative error relative to their own mean day than did females (CBT: *p* = 0.003, Fig. 4C; LA: *p* = 7 × 10^–4^, Fig. 4G). However, given that males also had higher variance overall, it could still be argued that the *proportional* amount of structured variance was the same, even if the absolute structure was lower. To account for this possibility, we divided the cumulative error values for each individual by that individual’s SD. The result confirmed that once the individual’s SD was corrected for, the amount of cumulative error was not different between the sexes (temperature: *p* = 1, Fig. 4C; activity: *p* = 0.24, Fig. 4H).

**Fig. 5:**
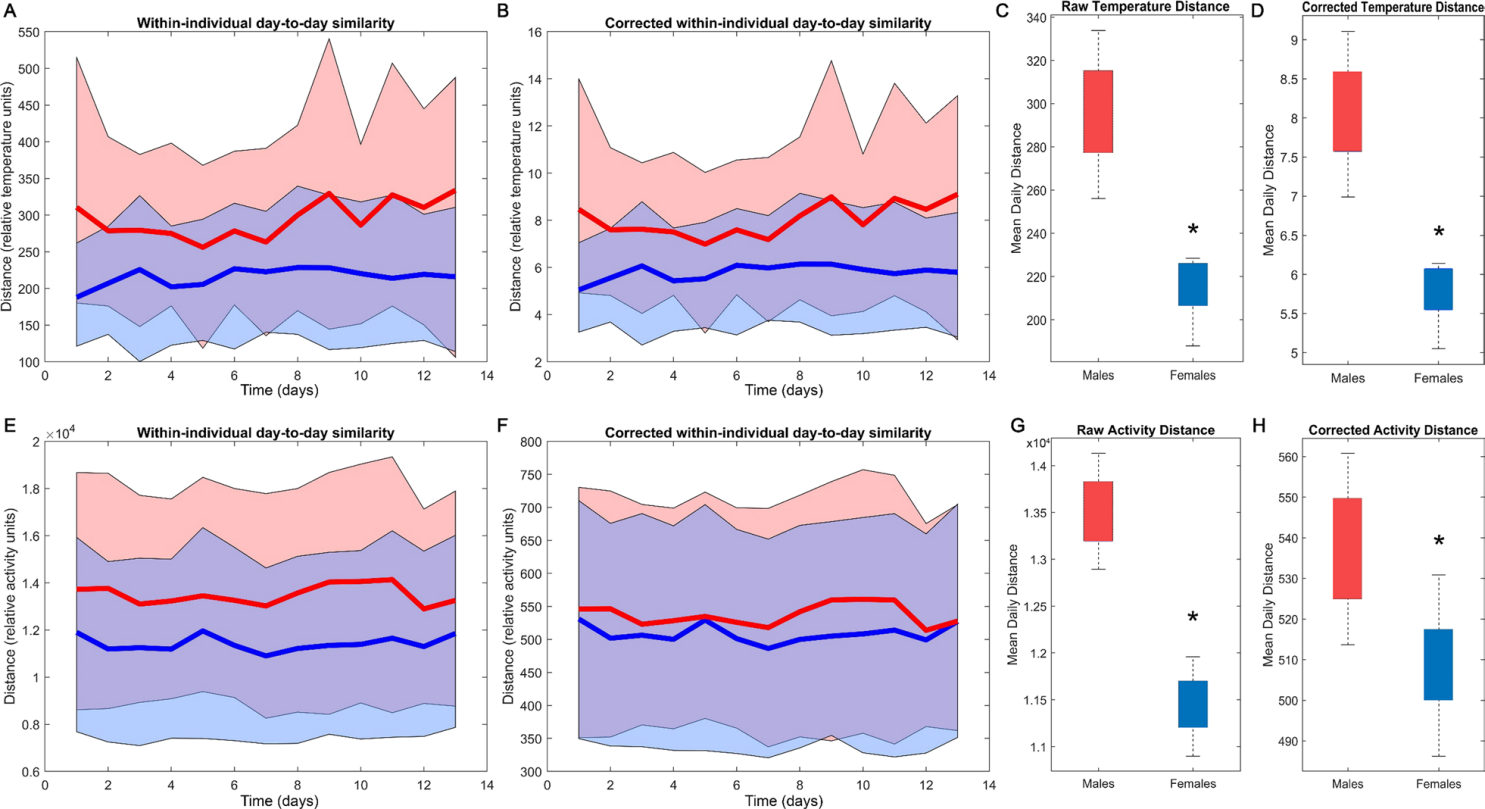
Males exhibit less day-to-day similarity than females. Mean and SD (thick line with shaded surround) of CBT **A**, **B** and LA **E**, **F** for males (red) and females (blue) of DTW distance between successive days. Males show higher within individual, day-to-day distance than females (**C**, **G**). This distance is slightly reduced but remains significant between the sexes when each individual’s distances are divided by their mean SD (**D**, **H**). This finding suggests that males had higher day-to-day variability within individuals, and less proportional structure, than females. *Indicates significant difference. See “Results” section for further details

We next examined within-a-day structure by comparing the distance needed (in units of activity or temperature, respectively) to warp one day to best match the subsequent day (see “Methods” section). By these analyses, males showed higher between-day distance for body temperature (Fig. 5A, D; *p* = 1.6 × 10^–5^) and also for activity (Fig. 5E, G; *p* = 6 × 10^–5^). As with analysis of daily means, distance is in the same units as variance, and so greater variance should cause higher average distance for the same proportion of structured variance in a given data set. As with the preceding analysis of comparison to average day, DTW distance calculated for each individual was corrected by dividing by that individual’s SD. This correction resulted in less distance between males and females, but males still exhibited a higher distance on average than females (Fig. 5B, D; temperature: *p* = 1.6 × 10^–5^; Fig. 5F, H; activity: *p* = 6 × 10^–4^). Because DTW is better able to align ultradian cycles with small day-to-day phase differences than is the daily mean, this finding more strongly supports the hypothesis that males are not only more variable, but also show a lower proportion of structured variance within the day.

## Discussion

By a number of different metrics and at several timescales, male mice were found to exhibit greater variability than female mice. This finding held true across days, within days, across individuals, and within individuals, even when females were staged to maximize inter-individual estrous phase misalignment. What’s more, females show a greater proportion of structured, compared to unstructured or unpredictable, variability at ultradian, circadian and infradian (ovulatory) timescales. By way of comparison to an average day, males have no advantage in proportional structure of variance within a day at ultradian timescales. By way of DTW (which captures ultradian similarity with greater fidelity, even if each cycle shows changes in precise phase alignment across days) males still had greater variability than females, even when correcting for their intrinsically higher overall variance, suggesting that within a day, less reduction of uncertainty by structure is possible for males than for females. Together, these findings suggest that a female mouse should be expected to generate substantially less error across random time samples than a male, and that if continuous data are captured to allow for contextualizing each measure at multiple timescales, then this female advantage should improve further. These findings are in stark contrast with the conventional wisdom that male mice are a better experimental choice because they are less variable than females for whom ovulatory cycle phase is unknown.

Here we show the importance of dynamic baselines for individuals and/or specific populations (here, sexes). Dynamic baseline comparisons successfully allowed an account of large proportions of overall variance from structured sources, especially at daily timescales where small changes make less of a difference in alignment across cycles, as was the case with ultradian timescales. With the emergence of wearable sensors generating time series data in human populations, the concept of the dynamic baseline could soon improve precision in physiological comparisons used in diagnoses; we made use of this assumption to improve fever detection during the first year of the COVID-19 pandemic [16], but to our knowledge, the type of numerical arguments presented in this manuscript have not been shown elsewhere explicitly.

Surprisingly, capturing estrous cycles in dynamic baselines contributed but only a small amount: 3% additional reduction when including estrous cycles in the dynamic baseline, compared to 30% reduction when including daily rhythms only. Whether this holds true for different physiological systems and for human data is an important direction for future examination, as it is the opposite of the common belief the estrous cycle is the source of a majority of structured variance. Indeed, since by many measures males showed greater within-a-day variance, this finding suggests that estrous cycles might be less of a concern when taking experimental measures than time-of-day assessment should be in male subjects.

Aligning ultradian rhythms in a way that makes their variance structured and predictable remains a challenge in need of better solutions; the difficulty of projecting structure onto ultradian rhythms may also suggest an underlying oscillator that is moderately variable and might reflect the output of a physiological dynamic equilibrium, as opposed to a circadian-style, more-tightly regulated oscillator. Further experiments are needed to directly assess these possibilities. With improved measurement and collection tools, ultradian rhythms might be more predictable across days, in which case the higher power of male variance at the ultradian timescale could make males a more appropriate choice for assessing difference between peaks and troughs of ultradian cycles. At present, the analyses presented here suggest that when mice are not tracked through time, females should be expected to generate lower variance in group or repeated measure analyses. It is also worth noting that DTW does not generate a distance metric per se, as DTW violates the triangle inequality in some cases. Nevertheless, there is robust literature demonstrating that DTW distance is an appropriate distance metric in the overwhelming majority of cases (e.g [17, 18].).

It is not clear from these analyses whether the effects revealed depend on strain, species, age, or variable measured. Much more work is needed to identify the variables and features the lend predictability to otherwise unaccounted for variance in biological measures, and to refine the concept of the dynamic baseline into a deployable form for research and clinical use. Such work is important for the sake of improving efficient use of animal subjects in research, for understanding how to improve precision and interpretation of point measures in diagnostics, and for overcoming the inertia of female-exclusionary beliefs that do not appear well founded and continue to disadvantage both female human and animal populations by hampering their inclusion in biomedical studies.

## Perspectives and significance

We do not find numerical support for the oft-cited claim that females are more variable than males. Female subjects have long been excluded from research, and biases are still frequently voiced to the effect that studying female models and or women is “too complicated,” implicitly referring to measurement variance introduced by the ovarian cycles of fertile females. Though not the only reason for biases in research, this cause lends itself to numerical testing. Using time series analyses on longitudinal data from male and female mice, we fail to support this commonly held hypothesis. Instead, we find that female mice show less overall variance, both within and across individuals, than do males, even without staging for the estrous cycle. We also find that there is less self-similarity in males than females across time, suggesting heterogeneity in both sexes should be further explored.

Females could be argued to be the safer choice of model organism, if one is forced to choose. Greater use of longitudinal measurement would allow reduction of experimental variance in either sex, and should be pursued when feasible. The day seems to be the major structured source of variance for both sexes, and ovarian cycles contribute much less than anticipated in mice. Our findings support the inclusion of Sex as a Biological Variable, rather than exclusion of any sex, when designing mouse physiological or behavioral research.

## Data Availability

All data and code are available upon request, and will be loaded to Dr. Smarr’s UCSD webpage upon publication (smarr.ucsd.edu).

## Abbreviations

CBT: Core body temperature
LA: Locomotor activity
SD: Standard deviation
CWT: Continuous wavelet transformation
DTW: Dynamic time warping
